# The associations between family interference with work and depression: the mediating role of stress and satisfaction in Saudi Arabia

**DOI:** 10.3389/fpsyg.2026.1920062

**Published:** 2026-08-19

**Authors:** Ahmed Muyidi, Xiaoxia Jojo Li, Yan Bing Zhang

**Affiliations:** 1 Department of Mass Communication, King Saud University, Riyadh, Saudi Arabia; 2 Department of Communication Studies, University of Kansas, Lawrence, KS, United States

**Keywords:** depression, family interference with work, family stress, job satisfaction, job stress, marital satisfaction, Saudi Arabia, work family conflict

## Abstract

Building on conservation of resources theory (Hobfoll, 2001) and spillover theory (Edwards and Rothbard, 2000; Staines, 1980), we conducted a survey study (*N* = 794) to examine the direct and indirect associations of family interference with work (FIW) through job stress, family stress, job satisfaction, and marital satisfaction on depression among dual earners in Saudi Arabia. The results indicated both time- and strain-based FIW were positive predictors of depression. Additionally, both job stress and family stress mediated the associations between FIW (time-, strain-, and behavior-based) and depression. However, marital satisfaction only mediated the relationship between strain-based FIW and depression, whereas job satisfaction was a nonsignificant mediator. Our study underscores the importance of family-to-work conflict and stress perceptions in impacting mental health. Theoretical and practical implications were further discussed.

## Introduction

Depression, referring to persistent low mood, reduced energy, and lack of interest in activities (World Health Organization, 2025) is a major common mental disorder affecting individuals’ wellbeing over the world. Specifically, 4.12% of the global population experience depression (Institute for Health Metrics and Evaluation, 2023). More seriously, the pooled estimate depression prevalence in Saudi Arabia was 37.35% (Nour et al., 2023). Additionally, major depressive disorder was among the top five causes of years lived with disability in Saudi Arabia in 2010 and 2017 (Tyrovolas et al., 2020). Beyond increasing humanistic concern about individual wellbeing, quality of life, and the physical and mental burdens experienced by individuals and their families, depression also places a significant economic strain on Saudi Arabian society. For example, healthcare expenditures and productivity losses in the Saudi workplace associated with mental disorders, including depression, are estimated to account for 4.1% of the nation’s gross domestic product (Arulsamy et al., 2025), which are higher than in the United States and Europe. Therefore, this study aims to explore factors and underlying mechanisms contributing to depressive disorders among Saudi Arabians.

Since the launch of the Saudi Vision 2030 initiative in 2016, Saudi Arabia has witnessed tremendous transformations from the traditional male-breadwinner and female non-working pattern to an increasing prevalence of dual-income households (Parveen, 2022; Muyidi et al., 2023; Zhang et al., 2023). It is therefore critically important to examine how these evolving family patterns influence the mental dynamics of dual-earners in Saudi Arabia, where the government has especially emphasized the importance for increasing women’s participation in the workplace (Zhang et al., 2023). Since family and work are the two major domains in adults’ lives, the current study focuses on the impacts of intersections of family and work on depression among Saudi Arabian dual earners.

This study applies conservation of resources theory (COR theory; Hobfoll, 2001) and spillover theory (Edwards and Rothbard, 2000; Staines, 1980) to explore family interference with work (FIW), which refers to conflicts arising when family life impedes work-related responsibilities (Greenhaus and Beutell, 1985; Michel et al., 2011). Among global studies of work–family conflict (WFC), defined as “a form of interrole conflict in which the role pressures from the work and family domains are mutually incompatible in some respect” (Greenhaus and Beutell, 1985, p. 77), the predictors, mechanisms, and outcomes of work interference with family (WIF), one direction of WFC, have been overwhelmingly examined. For instance, studies illustrated that work time predicted work inference family, which can in turn lead to psychological distress (Major et al., 2002). However, research on FIW as the focal predictor, the other direction of WFC, has received limited attention from researchers. Given that the two conflict directions are empirically distinct, demonstrating unique variance and differential patterns with external correlates (Mesmer-Magnus and Viswesvaran, 2005), examining FIW independently and its distinct mechanisms and outcomes is critical for advancing better understanding of work-family dynamics.

Although studies have consistently shown that work–family conflict, in both directions, is related to mental health, the mechanisms underpinning these relationships remain underexplored. More specifically, inadequate attention has been devoted to positive and negative factors across family and work domains as pathways through which FIW lead to adverse mental health outcomes (e.g., Wang and Peng, 2017). This study simultaneously applies the cross-domain principle to test work-related outcomes of FIW, including job stress and job satisfaction, and the matching-domain principle to examine family-related outcomes, including family stress and marital satisfaction (Amstad et al., 2011), investigating the extent to which these outcomes account for the relationship between FIW and depression. At the same time, the present study integrates both positive and negative factors in the family domain (i.e., family stress versus marital satisfaction) and in the workplace (i.e., job stress versus job satisfaction) as explanatory mechanisms in explicating Saudi adults’ depression.

Additionally, although FIW has been documented in various cultural contexts, such as Germany, Poland, and Ukraine, in recent years (Kuśnierz et al., 2022; Lange and Kayser, 2022), research on work–family conflict in Saudi Arabia remains underexplored. Among the limited studies on work–family conflict in the Saudi Arabian context, Alghanemi et al. (2025) found that depressive physicians reported higher levels of work–family conflict than their counterparts, while AlAteeq et al. (2025) found that 50.2% working women in Riyadh region had experienced mild to moderate levels of depression. To contribute to the existing literature, by examining FIW among Saudi Arabian dual-earners, this study can expand insights into work-family dynamics within the Saudi Arabian cultural context and enrich the global literature on work-family relationships.

### Family interference with work

Work–family conflict (WFC) comprises six distinct dimensions, reflecting the combination of two directions (i.e., work interference with family and family interference with work) and three forms (i.e., time-, strain-, and behavior-based conflict; Carlson et al., 2000). Time-based WFC occurs when time used for one role (work/family) cannot be applied to another role (family/work) (Greenhaus and Beutell, 1985; Hetrick et al., 2024). For instance, parents who need to pick up their children may have to take time off from work. Strain-based WFC arises when strain caused by one role affects people’s performance in another role. Stressors such as unsupportive family members can cause strain symptoms like depression, which undermines fulfillment in the workplace (Greenhaus and Beutell, 1985; Hetrick et al., 2024). Lastly, behavior-based conflict occurs when behavioral patterns expected in one role are incompatible with those expected in another role. For instance, men may be expected to be self-resilient and emotionally stable at work, while as family members, they are expected to be warm and emotional (Greenhaus and Beutell, 1985; Hetrick et al., 2024). In this study, we aim to explore these three forms of work–family conflict to obtain a comprehensive understanding of work–family conflict.

Regarding work–family conflict (WFC), scholars initially examined WFC unidirectionally, focusing on the conflict that occurs when work interferes with family (WIF; Carlson et al., 2000). Even though extant studies still predominantly explore WIF, researchers have increasingly held a consistent view that each of the three forms of WFC can combine with two directions: WIF, such as working overtime restraining people from accomplishing home duties, and family interference with work (FIW), such as taking care of family members preventing attendance at work. This study focuses on FIW as our focal independent variable.

### FIW and depression in Saudi Arabia

According to conservation of resources theory (COR theory, Hobfoll, 2001), stress occurs when people are threatened with resource loss, experience actual resource loss, or fail to gain enough to offset what they invest. Regarding the work and family dynamics, FIW is the process in which the demand in family drains time, energy, and emotional resources which can be used in the workplace (Sun et al., 2024). Stressors in the family domain and strain of navigating incompatible role expectations can impede satisfaction within family roles and in workplace (Ford et al., 2007). As anticipated or actual resource losses accumulate in family and/or work, individuals may become increasingly vulnerable to psychological strain, heightening the risk of mental suffering and related distress. COR theory offers a framework for understanding the outcomes of inter-role conflict stress resulting from possible or actual losses of resources in the process of shifting from roles in workplace and family and which in turn can lead to negative psychological or physiological states (Grandey and Cropanzano, 1999).

According to a recent report, 29.7% Saudi women in the third quarter of 2025 were employed (General Authority for Statistics, n.d), indicating the increasing labor force participation rates in Saudi Arabia (Parveen, 2022). The growing woman labor force participation reflects the increased presence of dual-earner households, which has not only transformed the landscape of work-family dynamics in Saudi Arabia but also given rise to related challenges. For instance, Alghanemi et al. (2025) found that higher scores of work–family conflict were associated with physicians’ report of depressive symptoms from the city Jeddah. From the cultural perspective, Saudi Arabia is a society with ingrained patriarchal norms, gender roles, and family obligations, which can challenge work-family balance and undermine mental well-being (Aldossari and Chaudhry, 2024). Additionally, existing research across different cultures has documented the associations between family interference with work (FIW) and mental health disorders. For instance, FIW was significantly and positively related to depression (Obidoa et al., 2011) in the United States. More recently, scholars found that, in Egyptian society, FIW predicted depression (Abdelrehim et al., 2023). Furthermore, Nasharudin and Rui (2024) found that all forms of WIF (time-, strain-, and behavior-based) for a spouse can lead to a teacher’s experience of FIW and in turn result in depressive symptoms. Based on previous findings of the potential effect of FIW on depressive disorder, we pose the following hypothesis:

*H1*: Family interference with work (H1a: time-based; H1b: strain-based; H1c: behavior-based) will positively predict depression.

### FIW and job stress, job satisfaction, family stress, and marital satisfaction

In acknowledging the ramifications of work family conflict (WFC), scholars have two distinct hypotheses: cross-domain versus matching domain (Amstad et al., 2011). The cross-domain hypothesis asserts WFC originating in one domain results in outcomes in another domain, whereas the matching-domain hypothesis contends that “the primary effect of work–family conflict lies in the domain where the conflict originates” (Amstad et al., 2011, p. 152). Applying these hypotheses to family interference with work (FIW), cross-domain hypothesis supports the effects of FIW on work-related outcomes. In contrast, matching-domain hypothesis assumes that FIW are more likely to link to family-related outcomes (Amstad et al., 2011).

This study simultaneously refers to the two hypotheses, which are supported by spillover theory (Staines, 1980) and extant studies (e.g., Ford et al., 2007) and can shed light on nuanced understandings to address extant inconsistent findings of these hypotheses (e.g., Zhang et al., 2020). Spillover theory asserts that individuals’ experiences in the family life can spread over to the workplace and vice versa (Staines, 1980) and, accordingly, scholars have conceptualized family-to-work conflict as negative family-to-work spillover (Stevens et al., 2007). The negative family-to-work spillover may increase job stress and undermine job satisfaction, which can spill over across domains to lead to adverse consequences, such as increasing family stress and lowering marital satisfaction.

Job stress and job satisfaction are two well-examined work-related outcomes. Job stress is employees’ physical and mental responses to the mismatch between job demands and the capabilities and resources available to them (National Institute for Occupational Safety and Health, 1999), while job satisfaction is people’s positive emotional state and affective reactions toward their working experiences (Oshagbemi, 1999). Empirical studies have found that FIW was positively associated with job stress while was negatively related to job satisfaction (Boles et al., 2001; Mansour and Tremblay, 2016). As regards family-related outcomes, two counterparts are family stress and marital satisfaction. Family stress is an imbalance between family demands and the capabilities or resources available to meet those demands (Mustafa et al., 2020), with family stressors manifesting in various forms, such as distributes between family members. Among scarce research on the relationship between FIW and family stress, one study found that family role conflict and ambiguity, as family stressors, were strongly positively related to FIW (Michel et al., 2010). Marital satisfaction is the perceptions of marital quality and the affective emotions of the marital status (Samadi et al., 2021). Existing research illustrated that FIW was negatively related to marital satisfaction (Bagherzadeh et al., 2016). Combining the cross-domain and matching domain hypotheses, along with extant empirical findings, we propose that FIW is associated with job stress and job satisfaction, as well as family stress and marital satisfaction among Saudia Arabian dual earners.

#### Mediating role of job stress and family stress

Encountering FIW can increase job stress and family stress as people struggle with incompatible demands across two domains. Empirical studies have supported the assumptions of COR theory to examine the associations among work family conflict, stress, and mental disorders. For instance, Hong et al. (2022) found that people with long working hours have higher risks of experiencing occupational stress, which bears a positive association with overall depression levels. Resulting from FIW, time pressures, emotional burdens, and juggling to behave in both family and work roles can serve as family and/or work stressors that encroach on the fulfillment of both roles and drain psychological energy (Dierdorff and Ellington, 2008). Expanding existing research, we suggest that all three forms of FIW (i.e., time-, strain-, and behavior-based; Carlson et al., 2000) can lead to both family and job stress, which can in turn increase depression. Thus, our hypotheses are:

*H2*: Job stress mediates the relationship between family interference with work and depression (*H2a*: time-based; *H2b*: strain-based; *H2c*: behavior-based).

*H3*: Family stress mediates the relationship between family interference with work and depression (*H3a*: time-based; *H3b*: strain-based; *H3c*: behavior-based).

#### Mediating role of marital satisfaction and job satisfaction

Drawing on the matching-domain hypothesis and the finding that FIW can lead to lower level of marital satisfaction (e.g., Bagherzadeh et al., 2016), as well as the negative association of satisfying marital function with depression (Trudel and Goldfarb, 2010), it is plausible to propose that marital satisfaction mediate the relationship between FIW and depression. Personal experiences in the family domain can spread over to the work domain (Staines, 1980). Family circumstances are responsible for the systematic patterns of negative family-to-work spillover (Crouter, 1984; Grzywacz and Marks, 2000), which can take the form of time-, strain-, or behavior-related interference with work. The negative spillover from family to work, including energy, attention, and mood (Crouter, 1984; Grzywacz and Marks, 2000), can lead to low job satisfaction, while job satisfaction was overwhelmingly examined to be strongly related to workers’ mental problems (Faragher et al., 2005). Therefore, our hypotheses are:

*H4*: Job satisfaction mediates the relationship between family interference with work and depression (*H4a*: time-based; *H4b*: strain-based; *H4c*: behavior-based).

*H5*: Marital satisfaction mediates the relationship between family interference with work and depression (*H5a*: time-based; *H5b*: strain-based; *H5c*: behavior-based).

## Method

### Participants and procedures

Data for the present study were collected from December 2024 to January 2025, following approval from the Institutional Review Board at a Saudi university in November 2024 through an online survey. Voluntary participants (*N* = 794; 419 men and 375 women) were recruited from Saudi Arabia to complete an anonymous online questionnaire in approximately 20 min. Specifically, participants were married and members of dual-earner families and were living with their spouses, with participation restricted to one individual per family to ensure independence of observations. They read the informed consent information presented on the first page of the online survey prior to participation. By proceeding with the survey, participants indicated their informed consent to participate and their responses were kept entirely confidential. Individual-level data are not shared publicly for protecting privacy and maintaining anonymity while de-identified aggregate data can be available from the authors upon reasonable request for academic validation.

The original questionnaire was in English and was translated into Arabic by an experienced bilingual Arabic expert who hold a Ph.D. in Communication Studies from the United States and back translated to English to insure its semantic validity of the translation in the Arabic cultural context. Participants’ mean age was 37.27 years old. The mean of years of work was 12.50 (*SD* = 9.29). The average length of marriage was 11.98 years (*SD* = 9.60), and the average number of children was 2.40 (*SD* = 1.98). Additional descriptive statistics for participants’ demographics are reported in Table 1. For the demographic variables, missing data were limited to two participants. One participant did not report the number of children, and another did not report years of work experience. For the major study measures administered after the demographic questions, we did not observe any missing data because the survey employed a forced-response design requiring participants to answer each item before proceeding to the next question.

**Table 1:** Demographic characteristics of the sample (*N* = 794).

Characteristics	n	%
Gender		
Man	419	52.8
Woman	375	47.2
Age range		
18–20	8	1.0
21–24	64	8.1
25–50	639	80.4
Above 50	83	10.5
Years of marriage		
1–10	430	54.2
11–20	218	27.4
21–30	118	14.9
Above 30	28	3.5
Number of children		
0	167	21.1
1–2	276	34.8
3–5	292	36.8
Above 5	58	7.3
Years of work experience		
1–10	399	50.3
11–20	235	29.6
21–30	126	15.9
Above 30	33	4.2

### Major measures

For the following major measures, we conducted a confirmatory factor analysis (CFA) with eight factors (time-, strain-, and behavior-based FIW, job stress, family stress, job satisfaction, marital satisfaction, and depression) with maximum likelihood estimation using RStudio (Version 2025.05.1 + 513) with the lavaan package (Rosseel, 2012). The model had a good fit to our data, χ^2^(791) = 1637.99, *p* < 0.001, RMSEA = 0.04, 90% CI [0.035, 0.039], SRMR = 0.04, CFI = 0.95, and TLI = 0.95, with standardized factor loadings for each item ranging from 0.70 to 0.91. For all confirmatory factor analyses, model fit was evaluated using the criteria recommended by Hu and Bentler (1999).

#### Family interference with work

We applied Carlson et al.’s (2000, p. 260) scale to measure family interference with work (FIW). Participants were asked to indicate their level of agreement on 3 items for each dimension of FIW (i.e., time-based, strain-based and behavior-based; 1 = strongly disagree and 5 = strongly agree). Higher scores indicate higher levels of conflict. For time-based FIW (e.g., “I have to miss work activities due to the amount of time I must spend on family responsibilities”; *M* = 2.65, *SD* = 0.92, *α* = 0.76). For strain-based FIW (e.g., “Because I am often stressed from family responsibilities, I have a hard time concentrating on my work”; *M* = 2.77, *SD* = 1.01, *α* = 0.87). For behavior-based FIW (e.g., “The behaviors that work for me at home do not seem to be effective at work”; *M* = 3.16, *SD* = 0.94, *α* = 0.89). Factor loadings for time-based FIW ranged from 0.70 to 0.74, and average variance extracted (AVE) was 0.52, while factor loadings for strain-based FIW and behavior-based FIW ranged from 0.76 to 0.89, and 0.82 to 0.88, respectively, with corresponding AVE being 0.69 and 0.72. All values of AVE exceeded 0.50, indicating adequate convergent validity.

#### Job stress

Job stress was measured with four items taken from the Motowidlo et al.’s (1986) scale of job stress. Participants were asked to indicate their degree of agreement on a six five-point Likert statements (1 = strongly disagree and 5 = strongly agree), about their stress with their current job (e.g., “I feel a great deal of stress because of my job”). Higher scores indicate higher levels of stress (*M* = 3.10, *SD* = 1.05, *α* = 0.91). The CFA showed that the factor loadings ranged from 0.82 to 0.88, and AVE was 0.71.

#### Family stress

Family stress was measured with four-item adapted from Motowidlo et al.’s (1986) scale to measure family stress at home. Participants were asked to indicate their degree of agreement on a six five-point Likert statements (1 = strongly disagree and 5 = strongly agree), about their stress within their home (e.g., “My family responsibilities are extremely stressful”). Higher scores will represent higher levels of family stress (*M* = 2.53, *SD* = 1.05, *α* = 0.92). The CFA illustrated that the factor loadings ranged from 0.79 to 0.91, and AVE was 0.73.

#### Job satisfaction

Job satisfaction was measured using six items adapted from two established scales: Brayfield and Rothe’s (1951) job satisfaction scale and the Cammann et al. (1983) measure. Participants were asked to indicate their level of agreement with the statement by selecting a number on a five-point Likert scale (1 = strongly disagree, 5 = strongly agree) about their satisfaction with their current job (e.g., “I find real enjoyment in my job”; “Most days I am enthusiastic about my job”; “Overall, I feel satisfied with my job”). Higher scores indicate high levels of job satisfaction (*M* = 3.29, *SD* = 0.94, *α* = 0.92). The CFA showed that the factor loadings ranged from 0.75 to 0.85, and AVE was 0.65.

#### Marital satisfaction

Marital satisfaction was measured with 10 items from ENRICH Marital Satisfaction Scale (Fowers and Olson, 1993). Participants were asked to indicate their level of agreement with the statement by selecting a number on a five-point Likert scale (1 = strongly disagree, 5 = strongly agree) about their satisfaction with their current spouse (e.g., “our relationship is a perfect success”; “I am very happy about how we make decisions and resolve conflicts”; “I have never regretted my relationship with my partner, not even for a moment”). Higher scores indicate high levels of marital satisfaction (*M* = 3.47, *SD* = 1.03, *α* = 0.97). The CFA reported that the factor loadings ranged from 0.78 to 0.91, and AVE was 0.75.

#### Depression

We used nine items from the PHQ-9 (Kroenke et al., 2001) to measure participants’ depressive symptoms (e.g., “Over the last 2 weeks, how often have you been bothered by any of the following problems?—“feeling down, depressed, or hopeless”; *M* = 0.96, *SD* = 0.78, *α* = 0.93). The CFA showed that the factor loadings ranged from 0.73 to 0.81, and AVE was 0.60.

## Results

### Data analysis procedures

Before we conducted the major analyses to test our hypotheses, we first examined Pearson’s correlations among the major variables (see Table 2). Following that, we used Model 4^1^ from PROCESS macro for SPSS (Hayes, 2018) with 5,000 Bootstraps to examine the direct effects (H1) and the indirect effects (H2–H5) of the three FIW variables through the four mediators (i.e., job stress, family stress, job satisfaction, and marital satisfaction) on depressive symptoms. Specifically, for each model estimation, depressive symptoms served as the dependent variable (i.e., the *Y* variable), one form of FIW (time-based, strain-based, or behavior-based) as the independent variable (i.e., the *X* variable), and job stress, family stress, job satisfaction, and marital satisfaction as parallel mediators (i.e., the *M* variables), while controlling the other two forms of FIW in addition to other covariates (i.e., age and gender). The direct and indirect paths between the focal predictors and the dependent variable were interpreted as significant if the 95% confidence interval (CI) did not contain a zero (see Table 3).

**Table 2:** Correlations among variables.

Variable	1	2	3	4	5	6	7	8	9	10
1. Age	–									
2. Gender (man = 1, woman = 2)	−0.13**	–								
3. TFIW	−0.05	0.02	–							
4. SFIW	−0.05	0.11**	0.58**	–						
5. BFIW	−0.05	0.05	0.36**	0.50**	–					
6. Job stress	−0.06	0.07*	0.53**	0.64**	0.46**	–				
7. Family stress	0.03	0.04	0.22**	0.30**	0.38**	0.37**	–			
8. Job satisfaction	0.12**	−0.04	−0.06	−0.05	−0.08**	−0.16**	−0.04	–		
9. Marital satisfaction	0.01	−0.15**	0.01	0.02	−0.07**	0.02	−0.28**	0.30**	–	
10. Depression	−0.14**	0.06	0.28**	0.33**	0.36**	0.40**	0.46**	−0.20**	−0.22**	–

**p < 0.01. *p < 0.05. TFIW, Time-based family interference with work; SFIW, Strain-based family interference with work, BFIW, Behavior-based family interference with work.

**Table 3:** Indirect effects of family interference with work (time-, strain-, and behavior-based) on depression through mediators.

Focal predictor	X ➔ M		M ➔ Y		X ➔ M ➔ Y	
	*b*	*SE*	*b*	*SE*	*b*	*SE*
Mediator 1: job stress (R^2^ = 0.30)						
TFIW	**0.31 [0.22, 0.39]**	0.04	**0.13[0.08, 0.19]**	0.03	**0.04[0.02, 0.06]**	0.01
SFIW	**0.20 [0.12, 0.27]**	0.04	**0.13[0.08, 0.19]**	0.03	**0.03[0.01, 0.04]**	0.01
BFIW	**0.26 [0.19, 0.33]**	0.04	**0.13[0.08, 0.19]**	0.03	**0.03[0.02, 0.05]**	0.01
Mediator 2: family stress (R^2^ = 0.31)						
TFIW	**0.22 [0.14, 0.30]**	0.04	**0.20[0.14, 0.25]**	0.03	**0.04[0.02, 0.07]**	0.01
SFIW	**0.39 [0.32, 0.47]**	0.04	**0.20[0.14, 0.25]**	0.03	**0.08[0.05, 0.11]**	0.02
BFIW	**0.11 [0.04, 0.18]**	0.04	**0.20[0.14, 0.25]**	0.03	**0.02[0.01, 0.04]**	0.01
Mediator 3: job satisfaction (R^2^ = 0.02)						
TFIW	−0.08[−0.16, 0.01]	0.04	**−0.08[−0.13, −0.03]**	0.03	0.01[−0.002, 0.02]	0.00
SFIW	−0.02[−0.10, 0.06]	0.04	**−0.08[−0.13, −0.03]**	0.03	0.00[−0.01, 0.01]	0.00
BFIW	−0.02[−0.05, 0.10]	0.04	**−0.08[−0.13, −0.03]**	0.03	−0.00[−0.01, 0.01]	0.00
Mediator 4: marital satisfaction (R^2^ = 0.04)						
TFIW	0.03[−0.07, 0.12]	0.05	**−0.08[−0.13, −0.03]**	0.03	−0.00[−0.01, 0.01]	0.00
SFIW	**−0.15[−0.24, −0.06]**	0.04	**−0.08[−0.13, −0.03]**	0.03	**0.01[0.002, 0.03]**	0.01
BFIW	0.04[−0.05, 0.12]	0.04	**−0.08[−0.13, −0.03]**	0.03	−0.00[−0.01, 0.004]	0.00

Significant effects (*p* < 0.05) are boldface. Unstandardized coefficients were reported as b. Bootstrapped 95% CIs are reported in brackets. TFIW, Time-based family interference with work, SFIW = Strain-based family. Interference with work, BFIW, Behavior-based family interference with work. R^2^ represents the proportion of variance in each mediator explained by FIW.

### Direct effects of FIW on depression: H1

Results indicated that both time-based FIW, *b* = 0.07, *SE* = 0.03, *p* = 0.03, 95% *CI* [0.01, 0.13], and strain-based FIW positively predicted depressive symptoms, *b* = 0.08, *SE* = 0.03, *p* = 0.01, 95% *CI* [0.02, 0.14], indicating that lack of working time and exhaustion generated from the family role that impedes performance in the workplace can lead to heightened levels of depression. Nevertheless, behavior-based FIW, *b* = 0.02, *SE* = 0.03, *p* = 0.30, 95% *CI* [−0.03, 0.08], was a nonsignificant predictor of depression among Saudi dual earners. Thus, H1a and H1b were supported, while H1c was not supported.

### Indirect effects of FIW on depression: H2 to H5

The findings (see Table 3 of all the indirect effects) illustrated that time-based family interference with work (FIW) was positively related to job stress and family stress, which in turn were positively related to depressive symptoms. These results mean that when spending time on their families with scarification of their worktime, Saudi Arabian dual earners may feel stressful in both family and work contexts, and in turn would be more depressed. Thus, H2a and H3a were supported. However, although both job satisfaction and marital satisfaction were significant negative predictors of depression, their associations with time-based FIW were nonsignificant. Hence, unlike what was predicted in H4a and H5a, the indirect effect of time-based FIW on depression through these mediators were nonsignificant (*p* > 0.05 for both, see Table 3).

Regarding the indirect effects of strain-based family interference with work on depression, except job satisfaction, the other three variables all worked as mediators for the relationship (see Table 3). The findings suggest that when being exhausted by the family role demands while hampering performance in the workplace, Saudi dual earners might experience heightened stress in both family and work contexts, lower levels of marital dissatisfaction, and in turn are more likely to experience depression. Therefore, H2b, H3b, H5b were supported while H4b was not supported.

Additionally, the results (see Table 3) indicated that both job stress and family stress explained the relationship between behavior-based family interference with work and Saudi dual earners’ depression. Nevertheless, the indirect path from behavior-based FIW to depression was not significantly mediated by neither job satisfaction nor marital satisfaction. These findings show that when people’s behaviors in their family life are incompatible with behavioral expectations in the workplace, they are more likely to be strained in both family and work contexts, which in turn heightens their susceptibility to depression. Thus, H2c and H3c were supported while H4c and H5c were not supported. In sum, job stress and family stress mediated the effects of all three forms of FIW on depression. However, marital satisfaction only minimally explained the link between strain-based FIW and depression, yet job satisfaction did not mediate the association between FIW and depression.

Overall, the FIW predictors and the mediators explained 33% of variance in depressive symptoms (*R^2^* = 0.33, *F*(9, 784) = 43.51, *p* < 0.001), indicating a large effect size (Cohen’s *f^2^* = 0.49; Cohen, 1988). Our findings provide meaningful empirical evidence that our model had strong explanatory power.

## Discussion

Motivated by the rise of dual-earner families under the Saudi Arabian government’s initiatives and the limited literature on family-to-work conflict among general populations in the Saudi context (Parveen, 2022; Muyidi et al., 2023), this study examined the associations among family interference with work (FIW), critical family and job variables, and mental sufferings for Saudia Arabian dual earners. To test Hypothesis 1, our results indicated that time-based and strain-based, but not behavior-based FIW directly predicted depressive symptoms. Time and pressure spillover from family to work can negatively impact people’s mental well-being. The significant effects of these two forms of FIW on depressive symptoms may be explained by individuals’ limited time and energy resources, as well as the co-existence of strain-based and time-based FIW, both of which hinder mental health (Charkhabi et al., 2016). In addition to exploring direct effects of the three FIW variables on depressive symptoms, this study shed lights on the mechanisms explaining the effects of work–family conflict on individuals’ mental health, through integrating factors in both work and family domains.

### Job stress and family stress as mediators

Based on conservation of resources theory (COR, Hobfoll, 2001) and spillover theory (Edwards and Rothbard, 2000; Staines, 1980), this study shows the mediating effects of job stress and family stress on the relationship between all three forms (i.e., time-, strain-, and behavior-based) of family interference with work (FIW) and depressive symptoms (in Hypotheses 2 and 3) among Saudi dual earners. These findings illustrated the processual dynamics that Saudia Arabian workers who encounter work family conflict originating from family spillover to work are more likely to experience stress across both family and work domains, which in turn increases their likelihood of experiencing depressive symptoms. In this process, as we suggested, FIW can have direct effects on both family-related outcome, family stress, and work-related outcome, job stress. In line with COR theory and spillover theory (Edwards and Rothbard, 2000; Hobfoll, 2001; Staines, 1980), FIW is negative spillover in which excessive physical and psychological demands on time, effort, and attention from the family domain, representing occurrent or potential resource losses, may lead individuals to perceive themselves as overwhelmed not only in family life but also in the workplace. Under family and work stress, people’s psychological functioning can be more vulnerable. Noticeably, the results were inconsistent with previous findings that WFC has stronger effects on same-domain outcomes than cross-domain ones (Amstad et al., 2011). We propose that the nature and intensity of work–family conflict contribute more to the resulting outcomes than whether the originating and receiving domains match. For instance, major life events, such as the passing of a partner or workplace bullying, can easily affect the same domain and spread their effects into other domains.

### Job satisfaction and marital satisfaction as mediators

Our findings indicated a nonsignificant mediating role of job satisfaction on the relationship between all forms of FIW and depressive symptoms (H4). The results illustrated that job satisfaction failed to explain the paths from family-to-work conflicts and mental disorders, despite the well-established relationship between job satisfaction and mental health (Faragher et al., 2005). Specifically, this study revealed a nonsignificant relationship between FIW and job satisfaction. This finding is consistent with the mixed evidence in the literature regarding the association between FIW and job satisfaction. For example, the study of Australian social workers illustrated that behavior-based FIW negatively predicted job satisfaction (Kalliath and Kalliath, 2015), while through a survey of probation and parole officers, Boles et al. (2001) found that FIW only weakly related to limited facets of job satisfaction, such as satisfaction with co-workers. The inconsistent findings in the existing literature suggest the possible multidimensional facets of job satisfaction and that the association between FIW and job satisfaction may be contingent on contextual or individual factors.

Additionally, this study illustrated partial support of H5 in that marital satisfaction mediated the relationship between strain-based FIW and depressive symptoms. Among the three FIW variables, the strain-based FIW significantly reduced marital satisfaction, which was in turn associated with more depressive symptoms. Overall. strain-based FIW was positively related to job stress and family stress and negatively related to marital satisfaction. This pattern of findings indicates that strain-based FIW may represent a pervasive stressor with its adverse effects extending across multiple life domains. Specifically, the psychological state of being overwhelmed or strain because of family responsibilities not only obstructs performances in the workplace among Saudi Arabian dual earners but also spills over into family and marital relationships. Consequently, among Saudi Arabian dual-earner employees, strain-based FIW may represent a cumulative manifestation of time-based and behavior-based FIW, which in turn negatively affects individuals’ well-being and marital outcomes, such as marital satisfaction. This finding is in line with Charkhabi et al. (2016) that strain-based FIW was the only significant predictor of hospital nurses’ mental health among all three forms of FIW.

Beyond these findings, we argue that spousal support may buffer the negative effects of strain-based FIW on depressive symptoms through marital satisfaction. Given its negative association with strain-based FIW (van Daalen et al., 2006), spousal support can help mitigate family-related burdens that hinder workplace functioning while simultaneously improving marital relationship, both of which may reduce depressive symptoms.

### Implications

Theoretically, our study expands the application of conservation of resources theory and spillover theory into interpreting the indirect effects of work family conflict on people’s mental well-being. Instead of focusing solely on family- or work-related outcomes of family interference with work, or on isolated psychological outcomes, this study identifies the pathways from work–family conflict through domain-related processes to general mental health outcomes. Our study offers new insights into the debate between the cross-domain and matching-domain hypotheses by suggesting the possibility that both mechanisms may coexist. We suggest that the originating domain of work–family conflict can synchronously have impacts the same and other domain. Additionally, we propose that matching-valence variables could exert more valid effects for the relationship between FIW and mental disorders. For instance, because FIW is a form of negative spillover, negatively valenced factors, such as job stress, were more likely to serve as mediators than positive factors, such as job satisfaction. Additionally, in contrast with the trend of examining work interference with family, our study verified the equal effects of FIW for interpreting individuals’ general mental well-being. Practically, this study highlights the importance of family life for employees’ perceptions, feelings, and performance in the workplace. Our findings inform policymakers on how to better address employees’ family demands and work-life balance through policies such as allowing for paid family leave and flexible working schedules to enable employees to maintain adequate family resources to sustain their work lives. Family members of employees should also consider sharing household responsibilities to enable them to engage in work effectively. Saudi employers need to acknowledge employees’ family concerns, reduce workloads, and endeavor to mitigate sources of work-life incompatibility. Practitioners can identify family-domain factors that lead to job stress, family stress, depression and implement appropriate interventions to improve individuals’ mental well-being. Lastly, individuals may cope with or adjust their perceptions of job stress and family stress strategically to reduce the impact of family issues on their mental health. For example, building reliable non-kin networks can be beneficial for dual-earners’ mental outcomes. Social support, such as support from friends, neighbors, and communities, may exert notable effects on the mechanisms underlying the relationships between FIW and mental disorders (French et al., 2018; Griggs et al., 2013).

### Limitations, future directions, and conclusion

This study centers on an underexplored direction of work–family conflict (WFC), family interference with work (FIW) together with both negative and positive domain-related outcomes for the underlying mediating mechanisms for linking FIW and mental disorders. Our findings indicated that FIW, stress and satisfaction outcomes in the family and work domains in our model accounted for a significant portion of variance (i.e., 33%) in depressive symptoms. Although this study examines a powerful model and offers several contributions, a few limitations need to be noted. First, approximately two-thirds of the variance in depressive symptoms remained unexplained, demonstrating that other variables not included in our model may also contribute to employee’s depressive symptoms. In addition to spousal support and workplace support, which we discussed earlier, conflict management styles between husbands and wives, both in general and during stressful times, are important factors associated with marital satisfaction, job satisfaction, and depressive symptoms (Lee et al., 2020; Song and Zhang, 2012). Future research should examine how conflict management styles and FIW jointly influence employees’ depressive symptoms. Other potential pathways for future research include the other, opposite direction of WFC (i.e., work interference with family) for explaining Saudi Arabian dual earners’ depressive disorder could provide additional insights. Future studies may incorporate both directions of WFC to provide a more comprehensive understanding of the relationships among WFC, domain-related outcomes, and mental health. Second, while this study was in response to the growing prevalence of dual-earner households in Saudi Arabia, future research may extend this project through conducting comparative studies between sole earner and dual earner households to shed light on how family-work dynamics impact Saudi individuals’ mental well-being. Third, this study did not examine the interdependent effects within intrafamily dynamics, future dyadic level studies among dual-earner couples can address this limitation (Vieira et al., 2018). Additionally, this study did not examine potential moderating effects, such as social support and endorsement of traditional norms, which can strengthen or attenuate the effects of FIW on mental disorders. Future scholars may explore these moderators to develop a more thorough understanding of social and individual factors that buffer or exacerbate the detrimental effects of work–family conflict.

More importantly, this study fills the gap of limited literature of family interference work (FIW) among general populations in the Middle Eastern societies, particularly amid Saudi government initiatives to expand workplace participation. Our findings reveal that FIW influences mental disorders (i.e., depression) both in direct and indirect ways through job stress and family stress. Additionally, being strained by family role while fulfilling work role could diminish marital satisfaction and lead to mental suffering. All these findings highlight the critical role of family life in work performance and mental well-being for Saudi dual earners.

## Data Availability

The datasets presented in this article are not readily available because individual raw data will not be shared publicly to maintain participant anonymity, but de-identified aggregate data can be made available upon reasonable request for academic validation. Requests to access the datasets should be directed to Ahmed Muyidi, amoaidy@ksu.edu.sa.

## Footnotes

1 Before testing our hypotheses, we used Model 8 to examine the potential moderated direct and indirect effects of FIW on depression. Results indicated that gender and age were nonsignificant moderators of any of the predicted effects. Hence, gender and age were only used as covariates in testing our hypotheses.
